# Recognizing, reporting and reducing the data curation debt of cohort studies

**DOI:** 10.1093/ije/dyaa087

**Published:** 2020-07-02

**Authors:** Oliver W Butters, Rebecca C Wilson, Paul R Burton

**Affiliations:** d1 Population Health Sciences Institute, Faculty of Medical Sciences, Newcastle University, Newcastle upon Tyne, UK; d2 Department of Public Health, Policy and Systems, University of Liverpool, UK; d3 Population Health Sciences Institute, Faculty of Medical Sciences, Newcastle University, Newcastle upon Tyne, UK

**Keywords:** Data curation, data management, cohort studies

## Abstract

Good data curation is integral to cohort studies, but it is not always done to a level necessary to ensure the longevity of the data a study holds. In this opinion paper, we introduce the concept of data curation debt*—*the data curation equivalent to the software engineering principle of technical debt. Using the context of UK cohort studies, we define data curation debt—describing examples and their potential impact. We highlight that accruing this debt can make it more difficult to use the data in the future. Additionally, the long-running nature of cohort studies means that interest is accrued on this debt and compounded over time—increasing the impact a debt could have on a study and its stakeholders. Primary causes of data curation debt are discussed across three categories: longevity of hardware, software and data formats; funding; and skills shortages. Based on cross-domain best practice, strategies to reduce the debt and preventive measures are proposed—with importance given to the recognition and transparent reporting of data curation debt. Describing the debt in this way, we encapsulate a multi-faceted issue in simple terms understandable by all cohort study stakeholders. Data curation debt is not only confined to the UK, but is an issue the international community must be aware of and address. This paper aims to stimulate a discussion between cohort studies and their stakeholders on how to address the issue of data curation debt. If data curation debt is left unchecked it could become impossible to use highly valued cohort study data, and ultimately represents an existential risk to studies themselves.

## Background

Software engineering has a well-defined concept of technical debt,^1^ first described in 1992.^2^ It gives an indication of the work required to re-engineer software when a suboptimal solution has been implemented. There are many reasons for intentionally accruing technical debt, for example time pressures or lack of available expertise. It is also common to accrue technical debt unknowingly, such as: when a software developer edits small parts of a large code base without understanding the overarching structure, they might introduce sub-optimal approaches. There are numerous ways technical debt can be incurred, including not documenting code when writing it or not depositing code in a version control repository.

**Key Messages**

- We introduce the concept of data curation debt - the data curation equivalent to the software engineering principle of technical debt.
- Data curation debt is common in UK and international cohort studies.
- Left unaddressed data curation debt has the potential to impact reproducibility and available resources in a study. Ultimately it can pose an existential threat to a study if it is not tackled.
- We outline a set of recommendations on how to address existing data curation debt, and reduce the accrual of new debt.

The use of the word debt is key here, since it can be thought of as analogous to a financial debt, which eventually must be paid back and accrues interest while it is left unpaid. The interest accrual on technical debt makes it more difficult to pay it back; this could be because as time passes it becomes more difficult to write documentation or commit the relevant code to a version control repository. It is important to note that even when steps are taken to change practice and stop the accrual of new technical debt, the existing debt still exists and must be addressed.

Often, the end users of software are not aware that a technical debt exists, and do not realize when it has been paid back. End users may benefit when this debt is paid off, as developers can spend less time maintaining their software and instead concentrate on building new features.

The UK is home to many cohort studies; the UK Medical Research Council lists 34 large (*N* >1000 at recruitment) cohort studies in their 2014 strategic review.^3^ These include studies that have been running for decades (e.g. the National Survey of Health and Development (NSHD) which started in 1946^4^). Outside this list exist many smaller cohort studies in the UK, likely to be in the hundreds in number.

Data-intensive subjects are increasingly adopting the FAIR data principles, which state that data should be Findable, Accessible, Interoperable, and Reusable.^5^ These principles have been followed in some domains for decades, although not explicitly labelled as ‘FAIR’. It is however only recently that the FAIR data principles have become the default stance of UK research funders across all domains.^6^ As cohort studies adopt the FAIR data principles it will give them increased visibility to researchers, and facilitate the adoption of new research methods (e.g. machine learning) as it becomes easier to link datasets together.

## Definition of data curation debt

In the context of cohort studies, where data have often been collected over a long period of time, we introduce an analogue of technical debt— “data curation debt.” We define data curation debt as the amount of work required to bring a dataset’s curation level up to a point that is acceptable to the study’s relevant stakeholders. Instead of suboptimal programming decisions causing the debt, it is the deviation from agreed good data management practices (see for example Corti *et al*.^7^) that causes the accrual of debt. Here an external researcher who is sent data from a cohort study is akin to a software end user in the technical debt scenario—they consume the data without much (or perhaps no) exposure to the data management practices of the study (i.e. how data are stored, how they are extracted etc.). The cohort study data manager is then analogous to the software developer.

Accruing a data curation debt makes it more difficult to use data in the long term. This could mean the data require more resource to make them available to researchers, which takes longer than it would if they were properly curated—i.e. if no data curation debt existed. Like technical and financial debt, data curation debt will accumulate interest, i.e. it becomes more difficult to pay back the debt as time elapses. If this debt is left unchecked it could become impossible to use the data at all if they become irrecoverably corrupt or lost entirely. If there is not a recognition that a data curation debt exists, then it is possible that partially corrupt data are being used in research projects. This might manifest as extra noise (e.g. incorrect pixels in a video) or missing (e.g. truncated) data which could lead to erroneous or misleading research findings. Like technical debt, there are reasons to knowingly accrue data curation debt, such as time pressures or limited availability of funding, as long as it is recognized that it must be addressed in the future.

## Examples and impact of data curation debt

Given our definition of data curation debt above, it is possible that different studies assign varying levels of debt to the same deviation from the ‘ideal’. For instance, whereas one study may strive to have all their data in an open format (as is the recommendation from Corti *et al.*^7^), a different study’s stakeholders may accept that all its data users are required to have proprietary software installed to open it, and therefore do not consider it a debt. With this reliance on the context of the data in mind, in Table 1 we describe some examples of common data curation debts of cohort studies which are a result of deviation from the ideal data curation scenarios. The ‘ideal’ that we refer to here is the scenario where all recommendations and guidance from Corti *et al.*^7^ are followed. In Table 1 we also highlight specific impacts each of the debts could have. The overarching impact is that it makes the data more difficult to use, or it is lost forever.

**Table 1.** Examples of data curation debt, the impact these may have on a cohort study or the ability to use the data, and possible solutions and preventive measures

Data curation debt	Impact	Possible solution and preventive measures
Data stored on legacy hardware (e.g. old unmaintained servers)	Legacy systems can break at any time and data irretrievably lost	Migrate data to maintained hardware and regularly review hardware lifecycle
Raw data stored on decaying physical media (e.g. drawings, consent etc. done on paper; interviews, behavioural tasks etc. stored on CDs/DVDs, MiniDisc, VHS, memory sticks, external hard drives etc.)	This type of information will often have been either completely transcribed or abstracted, but if the physical medium decays then the raw data are lost. This makes it impossible to reproduce the original research or apply new techniques to analyse the original raw data. In the case of paper consent decaying, this can lead to legal issues	Digitize raw data where appropriate. Move already digital data on external devices to maintained storage. Record a date by which physical media is due to have decayed in the study data asset register
Data stored in obsolete/proprietary data formats	Once data are in an obsolete or proprietary format they become difficult to use and process. Format conversion is required before such data can be transferred to researchers. This may not be possible or feasible, and the data may effectively be lost	Migrate data to open formats. Regularly review formats used and assess their continued use. Prioritize the use of open formats in new data collections
Data not properly structured (e.g. messy file trees with no indication of completeness)	A data manager may have to make a best guess as to what historical data are and how they should be structured in order to give them to researchers. The risk is that the data may be misinterpreted, be incomplete, not the latest version etc.	Restructure the data as soon as possible. Develop policy to ensure new datasets are properly structured
Little or no documentation or provenance information	Retrospectively writing documentation is inherently error prone. A data manager may have to make a best guess as to where the data came from and how it has been edited	Write documentation as soon as possible. The longer it is left, the more organizational memory will disappear. Develop policy to ensure new datasets must have documentation when created
Changing good data management practices not retrospectively applied (e.g. updated disclosure control methodologies)	Data which have been released with old disclosure control methodologies applied become more of a risk as re-identification techniques and technology evolve, and as other datasets become available	Regularly assess the risk to the study of not applying new data management practices retrospectively
Carelessly aggregating data from third parties (e.g. social media)	As third party data providers change their software (e.g. APIs) and terms and conditions are updated, the underlying data model may change. This can lead to extra data being erroneously collected for which explicit consent is not present, and may also affect the ability to reproduce a dataset	Where there is any doubt in the consent of third-party data, they must be deleted. When collecting new data from third parties, the relevant metadata (covering for example terms and conditions) need to be kept with the data

This list is not intended to be exhaustive.

The loss of data has far-reaching consequences: a recent survey of scientists highlighted that the lack of availability of methodology, software code and raw data were common factors driving irreproducible research.^8^ The accrual of data curation debt is a key contributing factor to raw data not being available and thus affects reproducible research. At a study level, the loss of data could lead to a loss of income—future grant applications would not be able to list it as a resource, or if the study charges for data then a revenue stream is halted. Even at a lower level, if data are not lost but require additional work to locate, tidy or format shift, then an additional staff cost is involved. The authors assert that many cohort studies in the UK are accruing data curation debt, either knowingly or unknowingly, with varying levels of impact on the studies.

## Causes of data curation debt

The FAIR data principles are used to different degrees across different domains. This variation is, at least partly, due to the differing governance conditions associated with the data in each domain—for example, in astronomy projects raw data are often deposited in publicly accessible repositories (e.g. the Hubble Space Telescope archive,^9^ the SuperWASP public archive,^10^ the Anglo-Australian Telescope archive^11^). This allows data on common objects (e.g. a star or a planet) to be catalogued, cross-referenced and made searchable to the public, with the raw data readily (and publicly) available. It is clearly not possible to do this with potentially identifiable individual level data on humans. This then means that in the medical sciences we have to implement extra steps such as: pseudonymizing or anonymizing data; using a data safe haven;^12^ or using some kind of managed data distribution process. It is the authors’ opinion that these necessary extra steps make the data management practices of cohort studies less transparent and scrutinizable by end users than in some other domains. This could be manifested by data being stored on legacy hardware or stored in a messy file tree etc., but since a data manager will collate (and perhaps pre-process) the data before a researcher receives them, the researcher is unaware of the original state of the data, i.e. the data curation debt. This has consequently fostered an environment where data curation debt can accrue unnoticed by cohort study stakeholders. The long-running nature of cohort studies greatly compounds the data curation debt interest and it can therefore have a much greater impact than in other domains.

Within this environment we believe the causes of data curation debt can be split into three main categories: longevity of hardware, software and data formats; funding; and a skills shortage.

###  

#### Longevity of hardware, software and data formats

Technology changes substantially over time frames much shorter than the expected length of many cohort studies, and this can have a huge impact on the hardware, software and data a study holds. Without careful monitoring and proactive management the hardware, software and data can quickly become obsolete. This includes hardware reaching its natural end of life, new versions of software being released that are not compatible with previous versions, or new data formats being used with no migration path from previous versions. In addition, software and hardware vendors often impose proprietary data formats—which then require a long-term commitment to licensing costs to view and work with the data. Add to this the risk of vendors becoming defunct, then there is a real risk to the ability to export historical data into modern open formats in the future.

#### Funding

Research funding cycles are usually short (often of the order of 1–5 active years), particularly so when compared with the length of time data are collected and used for research in a cohort study (e.g. the NSHD, which is over 70 years old). Research funding opportunities are also often science-driven and can be influenced by the priority research areas of the funders. This is at odds with the necessity for long-term strategic planning of data curation to ensure long-term data access,^13^ and also the requirement by some funders to keep data for long periods of time. For example, the UK’s research funding councils have common principles on data retention, recognizing that data required for validation, or data that cannot be re-measured (e.g. human observation data), may require permanent retention, regardless of whether it is used in publications. Data may, however, be discarded if the ability to validate published research findings is not compromised. Additionally, data underpinning research publications are expected to be accessible for 10 years after publication.^14^ Similarly, the Wellcome Trust good research practice guidelines require research data to be archived and accessible for a minimum of 10 years after the study ends. If the research is based on clinical samples or findings that relate to public health, it should be retained for 20 years.^15^ Charities funding biomedical research, such as Cancer Research UK, also require funded projects to make data available for at least 5 years after a project ends.^16^

Funding data curation and maintenance of historical data, beyond the support necessary to complete data access requests, is often not eligible or competitive for research funding.^13^ Even at a very basic (and quantifiable) level, essentials such as the costs of long-term data storage are difficult to fund—a data collection project that collects 100 TB of image data today may find it difficult to secure funding up front to pay for its storage for the next 20 years, therefore underestimating the true cost of the exercise.

In this funding environment, it is inevitable that some cohort studies will have no choice but to focus their funding applications on the short-term science goals of the funders, rather than their own long-term data curation requirements.

#### The skills shortage

A review commissioned by the Wellcome Trust revealed that a lack of data curation and management skills, as well as relevant training within the academic sector, is contributing to a skills shortage.^17^ This is closely linked to the difficulty of attracting and maintaining research software engineers or staff with the necessary technical skills to work in an academic setting where there is little job security,^18^ with short contract lengths, with roles often funded by cost recovery on research grants and with pay substantially less than non-academic equivalent roles. The lack of recognition of data curation as an essential research activity, coupled with the lack of professional status for data managers and research software engineers, mean they may also lack recognition within an academic setting. For example, a software Sustainability Institute Survey in 2016 found that 88% of UK research software engineer respondents contribute to results within academic publications, yet a quarter of those get no acknowledgement on the paper.^18^ The equivalent 2018 survey showed some improvement, with a fifth now saying they get no acknowledgement in papers.^19^ This is not the case for researchers contributing to results in publications, who typically get co-authorship. Consequently, there is limited career progression for research software engineers in academia, particularly if they are unable to generate the same quantity of metrics as researchers (e.g. publications and securing grant funding) which are used to judge promotion and progression in the sector.

The lack of recognition, and therefore career progression, of data managers also leads to a lack of data management authority in senior decision-making roles in cohort studies. This can compound a data curation debt, as those with the requisite knowledge and experience do not have the authority to make the decisions which ultimately address the data curation debt.

## Recognizing and reporting the debt

In order to address the data curation debt in a cohort study, the first step is a recognition that a debt exists and to quantify its current extent and impact. This is a non-trivial exercise and is likely best undertaken by an independent body outside the study, since the study may unknowingly be accruing debt or downplaying the extent of the debt for fear of reputational damage. The groups best suited to carry out this exercise are data managers in other cohorts, experts in large-scale hosting of cohort data such as the UK Data Service [ukdataservice.ac.uk] or digital curation experts like the Digital Curation Centre [www.dcc.ac.uk]. In order to engage an external body, funding will likely need to be sought or some kind of reciprocal agreement with other cohort studies made. In either case, the funding bodies will ultimately have to pay for it.

Like technical debt, and unlike financial debt, there is no easy (or meaningful) way to put a single figure on the amount of data curation debt a study holds. What can be done is to compare the existing state of data curation with the level the study and stakeholders want to achieve. Different studies will have different requirements based on their data—e.g. an imaging-focused study will be different from a survey-based study. A logical place to start is with the study’s data asset register, and to use this to catalogue the status of relevant data curation criteria for each dataset. This could include, for example, the current format of each data item the study owns—if it is an open format, or if a migration exercise is required. Integrity status could also be added. Does each data item have a checksum applied to it? When was the integrity of the data last checked? Do metadata exist for the data item? When was the last time disclosure control checks were performed on the data?

Including this information in a public-facing reporting mechanism would also increase the usefulness (and potentially the use) of the data. This could cover relevant attributes of a dataset such as: whether the format of the data, and the software (including version) required to open it, are open source or not; quality of the documentation; ease of access etc. This would make it easier to advertise data with a high data curation debt (e.g. video recordings stored on VHS) alongside readily available data by giving a suitable context. By doing this, potential users of the data would be able to more easily judge the usefulness of the data before applying for it (e.g. if a dataset requires a license or has no documentation then it may not be suitable for a given user).

## Reducing the debt

Eradicating a data curation debt may not be a realistic short-term goal, particularly in large and/or long-running studies. Once the extent of a data curation debt has been recognized a prioritization exercise can take place. This should focus on the risk of losing data, the impact the loss would have and the available resources to address it. The context of the data makes it impossible to prescribe specific actions that are relevant to all scenarios here. With this in mind we make some strategic recommendations that will help address existing data curation debt and will reduce the accrual of new data curation debt. These recommendations are based on a combination of guidelines and best practice from this domain,^3^^,^^7^^,^^17^ as well as non-domain specific principles^5^^,^^6^^,^^13^^,^^18^ and the authors' personal experiences of using cohort data, managing large UK cohort studies at various levels (including roles as principal investigator and co-investigator on several large UK birth cohorts, data manager, senior data manager) and positions on various committees on data management/access (e.g. Managing Ethico-social, Technical and Administrative issues in Data ACcess—METADAC,^20^ Expert Advisory Group on Data Access—EAGDA^21^), membership of data-driven organizations such as the Research Data Alliance [www.rd-alliance.org], Software Sustainability Institute [www.software.ac.uk], CLOSER [www.closer.ac.uk] etc.

- Have a strategic goal to identify and reduce data curation debt. This needs to come from the highest level of study management, ensuring there is a voice for the data managers at a high level in the organizational structure.
- Transparently report the level of data curation debt in the study to the relevant stakeholders regularly.
- At a study level, dedicated resource must be allocated to address data curation debt, and this should be an explicit work package when applying for relevant grants and included as part of the data management plan. With this necessary allocation of funds comes an opportunity cost of doing other important work.
- At a national level, the funding bodies need to recognize that investment is required to address these debts; moreover, we suggest that funding bodies should actively require cohort studies to itemize their data curation debt as a part of future applications for funding.
- Embed stakeholder-agreed good data management principles into the core of the study in order not to unintentionally accrue new debt. Guidance can be taken from sources such as Corti *et al*.^7^ in compliance with FAIR data management principles^5^ and necessary research funder requirements. However, we recognize that this is data context-specific.
- Mitigate the risk of vendor lock-in by moving to open and open-source hardware, software and data formats where specifications, designs and source code will remain available even if a project closes. An example of good practice is the accelerometers used by UK Biobank. Instead of using off-the-shelf proprietary models, which may have opaque algorithms in the data collection and processing, the project opted for a model that is open source, allowing fully transparent data analysis to be carried out from the raw data.^22^
- Be ready to take drastic decisions about poorly curated data—e.g. it may not be financially viable to digitize old cassette tapes, so accepting that they should be discarded, and the data destroyed needs to be a recognized option.
- Elevate the standing of curated data to a first-class research output in the organization. The UK's Research Excellence Framework already gives equal weight to software and research datasets as journal articles, meaning that organizations can submit well-curated data knowing it will be judged in parity with journal articles.^23^
- Where possible implement a system to ensure those involved in data curation are properly credited in outputs. A practical step in this is the use of the Contributor Roles Taxonomy [CRediT—www.casrai.org/credit.html].^24^
- Embrace and stay abreast of technology changes so data are not collected and stored on unsupported/obsolete platforms.
- Invest in the specific training of data managers and in the general understanding of the importance of research data management for principal investigators and management teams.
- Work with national consortia such as the CLOSER consortium [www.closer.ac.uk] to keep data managers up to date with what others in the field are doing.
- Provide resource to support data managers to engage with the wider research data management community—both within and external to the domain—to refresh knowledge and best practice. This may include attendance at meetings focused on data management, i.e. those of the Research Data Alliance [www.rd-alliance.org], CODATA [www.codata.org] and ICSU-WDS [www.icsu-wds.org].
- Develop consistent job descriptions and career paths for those involved in data curation. This is starting to be addressed for research software engineers involved in research projects, and the lessons learned there can be translated into data management and archiving roles.
- Make sure that remuneration of data curation staff is competitive with other sectors in order to attract and retain those with the required technical skills.

## International applicability

This opinion piece has been framed in a UK context to discuss the nature, impact and solutions to prevent and manage data curation debt. From an international perspective, the work presented here is important on two fronts. First, as contemporary cohort study research increasingly links data across geographical borders, it is important that researchers appreciate that they must consider the different contexts from which the data came. Second, the authors’ involvement in current, and past, international cohort study consortia (as well as international workshops and conferences) has highlighted that data curation debt is a common occurrence in cohort studies internationally. The causes may differ slightly in other countries (e.g. different funding council policies) but the end impact is the same. We are confident that the recommendations to reduce the debt outlined here are transferable to an international audience.

## Conclusion

In the cohort studies domain where the recruitment and maintenance of participants is so difficult, it is unacceptable that there is such a risk to the hugely valuable resource of historical data through the accrual of data curation debt. The causes of this accrual are well known, and have been for many years, but have not been systematically addressed.

The near ubiquitous inadequacy of infrastructure funding, and the constant need to demonstrate novel and high-quality scientific outputs to justify the limited funding that is available, compels investigators running cohort studies across the world to prioritize short-term scientific outputs over longer-term investment in the curation of their data. Unfortunately, this perspective can only succeed up to the point where growing flaws in the underlying data infrastructure start to threaten the scientific programme itself.

Unless substantial effort is made to pay back data curation debt, there is a very real possibility that some cohorts will no longer be financially viable, as they have to spend more of their resources paying the interest on the debt and never address the debt itself. The only way this can systematically change in the field is through a combination of honest, transparent reporting of the level of data curation debt, and a recognition from the funders that this is a vital issue that they must fund. Not addressing the data curation debt jeopardizes the original investment in the research, contributes to the reproducibility crisis and threatens the transparency of research in the domain.

## Funding

This work was supported by the Wellcome Trust and Medical Research Council (grant number 108439/Z/15/Z to O.B. and P.B.), the Economic and Social Research Council and Medical Research Council (grant number ES/K000357/1 to O.B. & R.W.), Department of Health (Connected Health Cities North East and North Cumbria to O.B. & P.B.), Medical Research Council (grant number MR/S003959/1 to R.W), the European Union’s Horizon 2020 research and innovation programme under grant agreement No 824989 and the Canadian Institutes of Health Research (CIHR) (O.B, R.W. and P.B.).

## Conflict of interest

None declared.
